# BUB1 and BUBR1: multifaceted kinases of the cell cycle

**DOI:** 10.1016/j.tibs.2010.08.004

**Published:** 2011-03

**Authors:** Victor M. Bolanos-Garcia, Tom L. Blundell

**Affiliations:** Department of Biochemistry, University of Cambridge, 80 Tennis Court Road, CB2 1GA. Cambridge, England

## Abstract

The multidomain protein kinases BUB1 and BUBR1 (Mad3 in yeast, worms and plants) are central components of the mitotic checkpoint for spindle assembly (SAC). This evolutionarily conserved and essential self-monitoring system of the eukaryotic cell cycle ensures the high fidelity of chromosome segregation by delaying the onset of anaphase until all chromosomes are properly bi-oriented on the mitotic spindle. Despite their amino acid sequence conservation and similar domain organization, BUB1 and BUBR1 perform different functions in the SAC. Recent structural information provides crucial molecular insights into the regulation and recognition of BUB1 and BUBR1, and a solid foundation to dissect the roles of these proteins in the control of chromosome segregation in normal and oncogenic cells.

## BUB1 and BUBR1 are versatile proteins

**Figure 1:**
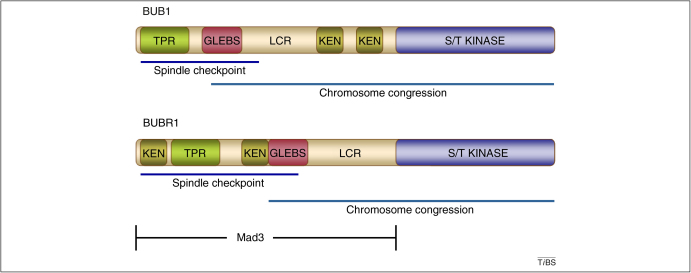
Domain organization of BUB1, BUBR1 and Mad3. The main functions associated with different domains are highlighted. The conserved functional motifs KEN, GLEBS and regions of low structural complexity (LCR) are indicated. Mad3 is a BUBR1 homolog that lacks the catalytic serine/threonine kinase domain.

The missegregation of sister chromatids during mitosis results in aneuploidy; the loss or gain of chromosomes in daughter cells. This disastrous outcome is avoided by the mitotic checkpoint for spindle assembly, also known as the spindle assembly checkpoint (SAC) (Box 1). BUB1 (budding uninhibited by benzimidazole 1) and BUBR1 (budding uninhibited by benzimidazole-related 1) also called BUB1B, and known as Mad3 (for mitotic-arrest deficient) in yeast, worms and plants) play central roles in this process. Three main regions can be identified in BUB1 and BUBR1 (two in Mad3): a conserved N-terminal region that contains the kinetochore localization domain; an intermediate, non-conserved region that is required for BUB3 binding; and a C-terminal region that contains a catalytic serine/threonine kinase domain (Figure 1). The BUBR1 homolog Mad3 lacks the C-terminal catalytic domain. However, there are no known species with both BUBR1 and Mad3; therefore, the functions fulfilled by BUBR1 in mammals probably are carried out by Mad3 in yeast, worms and plants.

Although they share a similar domain organization, BUB1 and BUBR1 (Mad3) are paralogs, and have distinct roles in the SAC. On the one hand, BUB1 is required for chromosome congression, kinetochore localization of MAD2, BUBR1 and centromere-associated protein (CENP)-E and CENP-F in cells with an unsatisfied mitotic checkpoint, and for the establishment and/or maintenance of efficient bipolar attachment to spindle microtubules [1–3]. Deletion of *bub1* from *Schizosaccharomyces pombe* increases the rate of chromosome missegregation [4], whereas deletion of *BUB1* from *Saccharomyces cerevisiae* results in slow growth and elevated chromosome loss [5]. On the other hand, BUBR1 associates with unattached/incorrectly attached kinetochores and plays roles in stabilizing kinetochore–microtubule attachment and in chromosome alignment [2]. BUBR1 forms part of the mitotic checkpoint complex (MCC) that also contains BUB3, MAD2 and cell division cycle 20 (CDC20), and inhibits the anaphase-promoting complex or cyclosome (APC/C) E3 ubiquitin ligase activity towards cyclin B1 and securin. Although there is considerable debate regarding the role of CDC20 ubiquitylation in the SAC (one model suggests that CDC20 ubiquitylation silences the SAC, whereas another suggests it exerts the SAC by degrading CDC20) [6,7], the mechanism of APC/C–CDC20 inhibition by the MCC points to a role for BUBR1 (Mad3) as a pseudo-substrate inhibitor. Moreover, BUBR1 regulates prophase I arrest, which is important for the progression through meiosis I to produce fertilizable eggs [8], and also accumulates to acentric chromatids that result from unrepaired DNA double-strand breaks [9].

Here, we review what is known of the domain organization and functions of the central mitotic checkpoint proteins BUB1 and BUBR1. We discuss the emerging evidence concerning 3D structures of the constituent domains and the contribution of such structural information for the molecular understanding of the roles of BUB1 and BUBR1 in the SAC. We also describe the mapping of cancer-associated and chromosome-segregation-deficient *BUB1* and *BUBR1* mutations onto existing structures. Finally, we consider how the structural characterization of higher-order complexes might provide further insights into the functional mechanism and regulation of the SAC signaling system as a whole.

## Functional insights from structural information

Considerable advances have been made in the structural biology of individual components and complexes of the SAC signaling pathway, including Mad2 (PDB codes: 1DUJ, 1KLQ, 1S2H, 2V64 and 2VFX), the Mad1–Mad2 core complex (1GO4), Mad2–p31(comet) (2QYF), Bub3 (1YFQ and 1U4C), Bub1 and Mad3 peptides mimicking the Gle2-binding-sequence (GLEBS) motif in complex with Bub3 (2I3S and 2I3T, respectively), as well as individual BUB1 and BUBR1 domains (Table 1).

### N-terminal region

**Figure 2:**
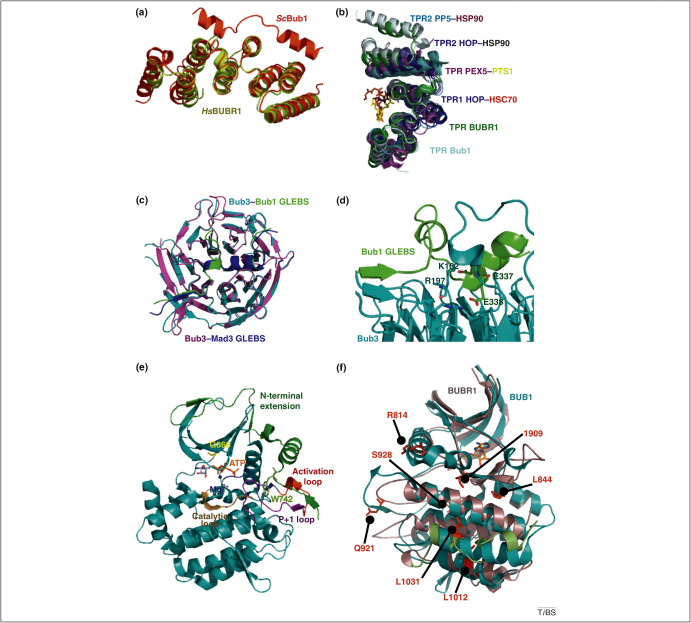
Structural understanding of BUB1 and BUBR1. (**a)** Superposition of the yeast (*Sc*) Bub1 TPR domain (red) and the human (*Hs*) BUBR1 TPR domain (yellow) showing the distinctive features of the “BUB fold”. (**b)** Superposition of *Hs*PP5 TPR (cyan), *Hs*HOP TPR1 (violet), *Hs*HOP TPR2 (deep blue), *Hs*PEX5 TPR (magenta), *Sc*Bub1 TPR (light blue), and *Hs*BubR1 TPR (green) reveals a similar concave face. PEX5 binds PTS1 (yellow), and HOP TPR1 binds the HSC70 peptide GPTIEEV (red). The PP5 TPR and HOP TPR bind the same pentapeptide sequence MEEVD (from HSP90, brown and black, respectively) in a different mode. In human TPR BUB1 and TPR BUBR1, residues of the blinkin-binding region map onto α helices equivalent to those of PP5 TPR, HOP TPR, and PEX5 TPR that are engaged in peptide binding, thus suggesting a similar binding mode. (**c)** Superposition of yeast BUB3–GLEBS complexes: Bub3 (magenta)–Mad3 GLEBS (deep blue) complex (PDB code: 2I3T), Bub3 (cyan)–Bub1 GLEBS (green) complex (PDB code: 2I3S). (**d)** Salt bridges formed between residue E382 of Mad3 (in Bub1 the equivalent position is E337) and Bub3 R-197 and between E383 of Mad3 (equivalent residue E338 in Bub1) with Bub3 K152 contribute to stabilization of the interactions. (**e)** Cartoon representation of the BUB1 kinase domain. The N-terminal extension and the P+1, activation and catalytic loops are highlighted. The magnesium ion is represented by the blue sphere. ATP, W742 (the residue that forms multiple contacts with the activation segment), and G866 (which functions as the gatekeeper residue) are shown in sticks. (**f)** Superposition of a 3D model structure of the kinase domain of BUBR1 and the crystal structure of BUB1 suggests the two domains exhibit high structural similarity.

The N-terminal domain, the most conserved region in both BUB1 and BUBR1 and their homologs [10], is essential for an efficient SAC [4]. Studies conducted in *Bub1* mutant murine embryonic fibroblasts have shown that deletion of two exons that encode a large part of the conserved N-terminal region leads to chromosome segregation errors, increased chromosome instability, and an attenuated spindle checkpoint response, thus confirming the importance of this region for BUB1 function [11]. Moreover, the N-terminal region mediates the physical contact of BUB1 and BUBR1 with blinkin. These interactions are important for the kinetochore localization of BUB1 and BUBR1 [12]. Furthermore, kinetochore-bound BUB1 is required for the recruitment of CENP-F, shugoshin (SGO1), and BUBR1 to kinetochores in cells with an unsatisfied mitotic checkpoint [13]. The crystal structures of the N-terminal regions of yeast Bub1 and human BUBR1, which are essential for binding blinkin, reveal a common fold that comprises a triple-tandem arrangement of the tetratricopeptide repeat (TPR) motif (Figure 2A) [14,15]. The TPR regions of human peroxin-5 (PEX5), heat shock protein 90 (HSP90) organizing protein (HOP), and protein phosphatase 5 (PP5) show an overall similar topology with the TPRs of BUB1 and BUBR1 (3.6 Å average rmsd of Cα) despite the low amino acid conservation in equivalent positions (Figure 2B). Features characteristic of TPR motifs and shared by Bub1 and BUBR1 include a concave inner surface and right-handed super-helical twist of the entire structure that results from the packing of α-helices. Bub1 and BUBR1 TPR tandems also adopt a unique BUB topology that is characterized by a shallow groove that is defined by a loop insertion in TPR1, an insertion of a 3_10_-helix between TPR2 and TPR3, and non-canonical packing interactions of TPR2. The significant reduction, but not elimination, of the interaction with blinkin after site-specific substitution of residues located within the groove suggests that kinetochore recruitment of BUB1 and BUBR1 involves several potentially co-operative interfaces. Future work is required to define the precise nature of these interactions and to establish whether the shallow blinkin-binding groove confers a unique functionality to N-terminal BUB1, BUBR1 or both.

The analysis of structurally related TPRs in complex with their peptide ligands provides some clues in this regard. Superposition of the crystal structures of the triple TPR tandem of Bub1 (PDB 3ESL) and BUBR1 (PDB 2WVI) with the TPR1 domain of HOP in complex with a heat shock cognate 70 (HSC70) peptide (PDB 1ELW); the TPR2A domain of HOP in complex with a HSP90 peptide (PDB 1elr) [16]; the PP5 TPR in complex with a HSP90 peptide (PDB 2BUG) [17] and the PEX5 TPR in complex with a peroxisomal targeting signal-1 (PTS1) peptide (PDB 1FCH) [18] show that BUB1 and BUBR1 residues involved in binding blinkin are mapped onto the TPR-forming helices that are involved in protein–peptide interactions (Figure 2B). Although the size of the protein–peptide interface in these complexes, measured as the change in solvent-accessible surface area buried on complex formation, is relatively small (HOP TPR–HSC70, 430 Å^2^; HOP TPR2–HSP90, 380 Å^2^; and PP5 TPR-HSP90, 260 Å^2^) and therefore unlikely to give tight binding, additional residues might contribute to the interactions in full-length proteins. For example, the binding of the HSP90 MEEVD sequence to HOP TPR2 induces a conformational change that seems to expose further interaction areas within full-length HOP [19]. It will be important to establish if similar gross conformational changes might occur during the interaction of blinkin with full-length BUB1 and/or BUBR1.

The KEN box, a protein motif defined by consecutive lysine, glutamate, and asparagine residues, that often mediates substrate recognition, is present in BUB1, BUBR1 and Mad3 (Figure 1). Two KEN boxes located in the N-terminal half of human BUB1 directly bind and ensure efficient phosphorylation of CDC20 [20]. Deletion of either KEN box affects BUB1–CDC20 binding, whereas deletion of both KEN boxes abolishes CDC20 binding. Consequently, the two KEN boxes are required for BUB1 ubiquitylation by APC/C–CDH1 [21] (CDH1 is responsible for APC/C activity from late mitosis to the G1–S transition). Flies that express BubR1 that harbors alanine substitutions of two residues, K7 and E8, which form part of the single N-terminal KEN box, exhibit a normal spindle function, but a defective mitotic checkpoint, thus indicating that the N-terminal KEN box is crucial for SAC function [22]. Although mouse BUBR1 contains one CDC20 binding site between residues 490 and 560, mutations that disrupt CDC20 binding to this region do not affect checkpoint function [23]. This observation is in conflict with a similar study based on truncated constructs of mouse BUBR1, where it is suggested that both KEN boxes are required for association with CDC20 [24]. However, an independent study based on the use of full-length BUBR1 and peptides that mimic the two KEN box motifs has shown that the N-terminal, but not the C-terminal KEN-box interacts directly with CDC20. Similar features have been observed in budding and fission yeast Mad3 [25–27], thus supporting the notion of BUBR1 acting as a competitive inhibitor of substrate binding in a process that is mediated by the interaction between the N-terminal KEN box and CDC20. Biophysical studies of N-terminal BUBR1 have demonstrated that the first BUBR1 KEN box is located within a flexible region of low complexity that extends from the TPR domain [15]. In addition to the low conformational constraints in this region, which should facilitate the presentation of the KEN box to APC/C–CDC20, diverse post-translational modifications might provide an additional level of regulation. For instance, a phosphoproteome analysis of cultured cancer cells has identified phosphorylation of BUBR1 residue T54 [28]. Although a role for T54 phosphorylation in mitosis remains to be established, this residue lies at the boundary between the N-terminal extension and the TPR domain [15]. This gives rise to the possibility of local conformational changes that affect the interaction of the KEN box and the APC/C-CDC20 complex. Moreover, BUBR1 undergoes acetylation by the histone acetyltransferase PCAF (p300/CBP-associated factor); K250 acetylation regulates APC/C-CDC20-mediated pre-anaphase degradation of BUBR1, thus contributing to the modulation of mitotic timing [29]. Given the importance of the already-characterized BUBR1 post-translational modifications, the identification and characterization of other BUBR1 post-translational modifications and their effects on the modulation of APC/C activity and mitotic timing are of utmost importance.

### BUB3 binding motif

Most of the residues that connect the N- and C-terminal regions of BUB1, BUBR1 and Mad3 are predicted to be mainly disordered. This region contains a conserved stretch of about 40 amino acid residues that is identified as the BUB3 binding site, and is commonly referred to as the GLEBS motif (residues 240–280 in human BUB1; 400–440 in human BUBR1 and 360–400 in *S. cerevisiae* Mad3). Overexpression of the GLEBS motif in HeLa cells causes disruption of the SAC by competing with endogenous BUB1 and BUBR1 for binding BUB3 [30]. Similarly, mutations that affect the GLEBS motif of human BUBR1 abolish the interaction with BUB3, an interaction that is essential for BUBR1 kinetochore localization [27]. The BUB1 and BUBR1 GLEBS motifs exhibit high amino acid sequence similarity with the GLEBS motifs of the human and yeast nucleoporins, NUP98 and Nup116, respectively. However, mouse BUB3 lacks affinity for the GLEBS motif of human nucleoporin NUP98, which demonstrates that the binding of SAC proteins to GLEBS motifs is highly specific. BUB3 is a protein that is organized as a seven-bladed β-propeller with a canonical WD40 repeat fold. The crystal structures of two independent complexes formed between yeast Bub3 and peptides that mimic the GLEBS motifs of Mad3 and yeast Bub1 reveal some conserved interactions (Figure 2C) [31], that is, a pattern of salt bridges formed between Bub3 and two glutamate residues of the GLEBS motifs (Figure 2D). In the two binary complexes, the GLEBS peptides form an extensive interface along the top surface of the β-propeller of Bub3. A single amino acid substitution in the GLEBS motif and the top face of Bub3 is sufficient to disrupt the protein interface, thus leading to extensive defects in chromosome segregation. The GLEBS motif–Bub3 interaction involves a transition from a predominantly disordered (unbound) to a more ordered (Bub3-bound) state. In view of the fact that regions of low structural complexity can bind to a wide variety of structurally distinct substrates, it will be interesting to define the role in ligand binding, if any, of conserved residues that surround the GLEBS motif.

## Kinase domain

Protein phosphorylation and dephosphorylation are important regulatory mechanisms of SAC signaling [32,33]. Nevertheless, the requirement of BUB1 and BUBR1 kinase activity in the mitotic checkpoint and in the stabilization of correct kinetochore–microtubule attachments remain contentious issues [24,30,34–40]. Some reports have suggested that BUB1 catalytic activity is of paramount importance because BUB1-mediated CDC20 phosphorylation inhibits APC/C–CDC20 in human cells [20]. Moreover, BUB1 depletion or expression of a BUB1 kinase-inactive mutant abolishes CDC20 phosphorylation and suppresses the SAC [20]. Independent observations that the BUB1 kinase-dead mutant is less effective in rescuing the defect of BUB1 knockdown [22] lend further support to the notion that BUB1 kinase activity is required for an appropriate SAC response. However, other reports have suggested that BUB1 kinase activity is of marginal relevance in the establishment of mitotic arrest [5,13,38], but instead, is important for chromosome alignment [13] and centromeric localization of SGO1, a protein that acts as protector of centromeric cohesion [41,42].

Phosphorylation and dephosphorylation of SAC components involve a complex signaling cascade [32]. BUBR1 undergoes auto-phosphorylation when the SAC is unsatisfied and acts as the substrate of other kinases such as Polo-like kinase 1 and cyclin-dependent kinase 1 (CDK1) [37,43]. Although some studies have shown that BUBR1 can inhibit the APC/C even after introduction of site-specific or deletion mutations that inactivate the kinase domain [44], others have concluded that BUBR1 kinase activity is crucial in this process [34]. Similarly, BUBR1 kinase activity might be important for efficient chromosome capture and congression [36,45]; however, other reports have indicated that BUBR1 kinase inactivation has a minimal effect on chromosome attachment [24,46]. Furthermore, prolonged mitotic arrest is triggered by BUBR1 kinase activity and/or phosphorylation [24]. Chromosome congression delay and unstable metaphase alignments have been observed in *Drosophila melanogaster* that expresses a kinase-dead BubR1 mutant (K1204A), thus indicating that BubR1 catalytic activity is required for correct kinetochore–microtubule attachments in flies [22]. Chromosome segregation is relatively unaffected in BubR1 kinase-dead mutant flies, which suggests that BubR1 kinase activity is substantially more important for spindle assembly than it is for the SAC.

Plausible explanations for the conflicting data on the role of the BUB1 and BUBR1 kinases in the SAC include intrinsic variations due to different assays used for measurement of SAC response and/or different efficiencies of depleting the endogenous protein [22]. In addition, the importance of kinase activity of mitotic checkpoint kinases might depend on the underlying genetic background and its inherent defects [13,32].

Although the role of BUB1 kinase activity in the SAC remains uncertain, clues about human BUB1 kinase regulation are provided by the crystal structure of its C-terminal region, which reveals a typical kinase fold (residues 784–1085) and an N-terminal extension (residues 724–783) that is highly conserved among BUB1 proteins and wraps around the kinase N lobe (Figure 2E). A particularly interesting feature is the interaction of the N-terminal extension with the kinase domain, which resembles the mechanism of activation of CDKs by cyclins. Although the crystal structure suggests a kinase in its active conformation, the C-terminal half of the P+1 loop adopts a hairpin-like structure that hides the catalytic loop, thus limiting access of ATP [47]. Such conformational features suggest that the structural reorganization of the C-terminal half of the P+1 loop (residues 965–972) is required for the efficient phosphorylation of BUB1 substrates.

A 3D model structure of C-terminal BUBR1 (residues 764–1044) shows the canonical features of a protein kinase: an N-terminal lobe that consists of a series of antiparallel β-sheets and a conserved α-helix, and a C-terminal lobe that is predominantly helical. A cavity located between the two lobes defines the ATP binding site. Superposition of a 3D model structure of BUBR1 kinase generated by comparative modelling using the crystal structure of BUB1 kinase as a template suggests that the two kinases adopt a very similar structure (Figure 2F). It is important to confirm this prediction and to determine whether an equivalent N-terminal extension that wraps around the kinase N lobe to modify the catalytic activity exists in BUBR1.

## *BUB1* and *BUBR1* mutations in cancer

Aneuploidy is a common characteristic among cancer cells. Deletions, insertions and point and silent mutations associated with aneuploidy, chromosome instability and cancer occur throughout the *BUB1* and *BUBR1* sequences (Tables 2 and 3). A role for BUB1 in oncogenesis is indicated by the occurrence of *BUB1* mutations, differential *BUB1* gene and protein expression in cancer tissues and cell lines, and the formation of spontaneous cancers in mice that express hypomorphic alleles [48–58]. *BUBR1* truncating and missense mutations have been identified in families with mosaic-variegated aneuploidy (MVA), a syndrome that is characterized by microcephaly and growth and mental retardation [40,59]. These biallelic mutations have provided new clues about cancer development; they are the first to relate germline mutations in a spindle checkpoint gene with a human disorder. Genetic testing has suggested that a decrease of >50% in BUBR1 expression (or activity) accounts for premature chromatid separation (PCS) syndrome in a cohort of Japanese families with monoallelic *BUBR1* mutations [60]. Moreover, aneuploidy and gastric cancer progression are known outcomes of BUBR1 overexpression [61,62]. Importantly, it might be possible to use BUBR1 expression as a marker of poor survival in certain types of human cancer [62,63].

Structural information allows the mapping onto the protein surface of some of the *BUB1* and *BUBR1* mutations that have been associated with chromosome instability and cancer progression. BUB1 residue A130 is exposed on the surface and lies in a region that connects the α-helices of the TPR units [14]. A substitution of this residue to serine (A130S) has been implicated in lymph node metastasis [53] and is predicted to disrupt stabilizing interactions between TPR-forming α-helices [14]. Recent evidence has shown that the A130S mutant impairs the localization of BUB1 at kinetochores, increases the rate of congression errors, and causes the loss of kinetochore binding of BUBR1, CENP-F and SGO1 [13]. The BUB1 H151D substitution probably has a similar impact on the structure, whereas the deletion mutant Δ76-141 should lead to a considerable disturbance of the entire TPR-containing domain structure [14]. The BUB1 substitution R209Q is mapped immediately after the TPR domain and onto a region that is predicted to be of low structural complexity. The effect of the R209Q substitution on the structure of BUB1 remains unknown. Quantitative immunofluorescence studies of two BUB1 substitutions, Y259C and H265N, which lie in close proximity to the BUB3-binding domain [53,54], show kinetochore localization and expression at levels comparable to native BUB1. The H265N substitution shows a normal chromosome alignment and spindle checkpoint and is able to recruit BUBR1, MAD1 and MAD2 to kinetochores in cells depleted of endogenous BUB1 [13]. By contrast, the BUB1 Y259C substitution does not rescue the spindle checkpoint. However, it efficiently restores chromosome congression and rescues the ability of kinetochores to bind SGO1 and CENP-F [13]. Further work is needed to clarify the mechanism of action of these mutations. Nearly 50% of the BUB1 substitutions associated with cancer can be mapped onto regions that are predicted to be mostly disordered (Table 2), thereby opening the possibility that substitution of these residues impairs protein–protein interactions.

Several cancer-associated mutations are found throughout the human *BUBR1* sequence. The Y155C mutant is mapped onto the third TPR repeat of the blinkin-binding domain. Y155C and R224 nonsense substitutions are associated with MVA and PCS syndrome, respectively. The E166D mutant, which has been identified in adult T-cell leukaemia/lymphoma (ATLL) patients, is mapped onto the loop region that connects α-helices A and B of the third TPR repeat. BUBR1 residue E166 is surface-exposed and highly conserved across species, which suggests a functional role. Interestingly, the majority of *BUBR1* mutations that are associated with different classes of cancer can be mapped onto regions of predicted low structural complexity (Table 3). Those mapped onto the kinase domain are the second most frequent, followed by those located in the blinkin-binding domain (Table 3). Several cancer-related mutants can be mapped onto the 3D model structure of the BUBR1 C terminus (residues 764–1044) (Figure 2F). The L844F mutant maps onto α helix αD; Q921H and S928 nonsense substitutions are located in the substrate-binding P+1 loop, and R814H maps onto the αC helix; a region that in BUB1 (and possibly BUBR1) enables ATP binding by neighboring residues [47]. Residue I909 is mapped immediately upstream of the magnesium-binding loop, whereas residues L1012 and L1031 map onto α helices αG and αH, respectively. Deletion of residues 1024–1050 is associated with colorectal cancer and results in a truncated protein that lacks α helices αH and αI. Substitution of L1012 by proline, which has been linked to hypothyroidism and anemia rather than cancer [40,59], is expected to affect the conformation of αG helix through the insertion of a kink at this position. Compared to *BUB1,* a larger number of *BUBR1* mutations associated with cancer have been reported to date. As noted above for BUB1, nearly half of the cancer-associated substitutions are mapped onto regions that are predicted to be of low structural complexity. It is particularly interesting to note that all the BUBR1 substitutions located adjacent to or within the kinase domain result in a severe decrease of protein concentration, probably reflecting an effect on protein stability [40]. In fact, the diverse substitutions mapped onto this region should affect protein stability to a different extent, thus resulting in a distinct decrease of BUBR1 concentration, a scenario that is consistent with the observed dependence of the amount of chromosome segregation defects on BUBR1 concentration in the cell [40]. Although studies on BUB1 and BUBR1 mutants have suggested that cancer formation is linked to a weakened SAC, the precise roles of these mutants in tumor formation remain unclear.

Changes in expression profiles of BUB1 and BUBR1 are often encountered in cancer cells and result in the impairment of mitotic checkpoint function. The observation that the weakening of the SAC provides an advantage for cell survival suggests that targeting the SAC might be a fruitful strategy for clinical anticancer therapies [64,65]. The crystal structure of the N-terminal domain of Bub1 reveals a hydrophobic pocket that binds CHES (2-[n-cyclohexylamino]ethane sulfonic acid), a compound of low molecular mass; this could be a useful site to target in drug discovery. The kinase N lobe, which is important for the regulation of BUB1 catalytic activity, binds the small molecule 2OH-BNPP1 (2-({4-amino-1-tert-butyl-1H-pyrazolo[3,4-d]pyrimidin-3- yl}methyl)phenol) in the ATP pocket with high specificity, and inhibits its activity. Hence, 2OH-BNPP1 and related molecules could constitute novel chemical toolkits for the study of BUB1 function *in vivo* and *in vitro*. More recent work has shown that pharicin A, a natural diterpenoid, which might act as an ATP-competitive inhibitor [66], can induce mitotic arrest of paclitaxel-sensitive and -resistant tumor cells through the inhibition of BUBR1 activity [66]. Whether these molecules can assist the design of compounds of potential therapeutic interest remains to be seen.

## Concluding remarks

The engagement of BUB1 and BUBR1 (Mad3) in multiple protein–protein interactions highlights their remarkable plasticity. The emerging structural details of BUB1 and BUBR1 (Mad3) provide a foundation for defining their functions in the SAC (Box 2); these details provide molecular insight into the recognition mechanism that mediates their localization to the kinetochore and the role of the amino acid residue substitutions that have been associated with cancer. Furthermore, given the potential benefit of targeting the SAC as a novel approach in anticancer therapy, the BUB1 and BUBR1 structures should be important in structure-guided drug design and the development of animal models that harbor cancer-derived mutations. Future work could aim to differentiate further the roles of the various domains and cellular pools of BUB1 and BUBR1 (Mad3) in the SAC and to elucidate the molecular mechanisms that mediate communication between the mitotic checkpoint pathway with the kinetochore–microtubule network and the DNA repair machinery. Atomic-resolution structures of most of the SAC protein components (alone and as part of macromolecular assemblies) should provide molecular insights into the mechanisms of regulation of the SAC and an understanding of how the spindle checkpoint translates the imbalance of force at the kinetochores into an APC/C-inhibitory signal.

## Figures and Tables

**Figure I:**
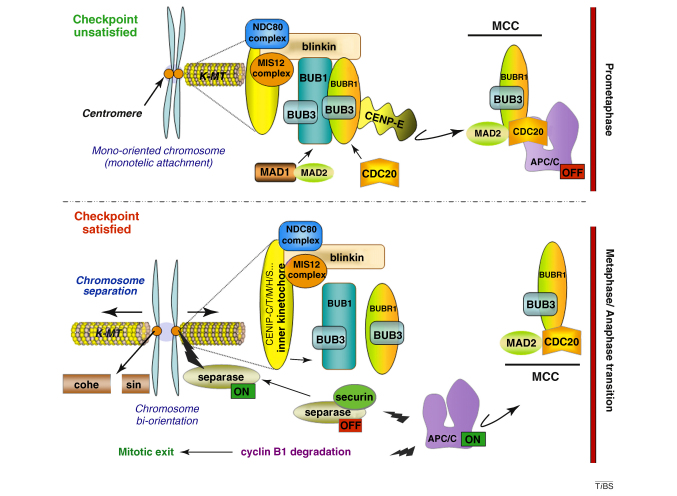
Simplified model of BUB1 and BUBR1 functions in the SAC.

**Figure I:**
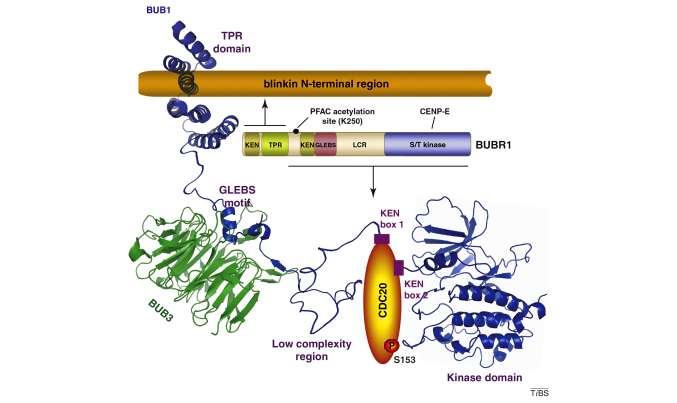
BUB1 and BUBR1 exert functions in the SAC through a range of protein–protein interactions.

**Table 1:** Crystal structures of BUB1 and BUBR1 domains reported to date

Structure	Organism	Construct length	Resolution	PDB code	Refs
Individual domains					
N-terminal Bub1	*S. cerevisiae*	29-230	1.74 Å	3ESL	[14]
N-terminal BUBR1	*Homo sapiens*	57-220	1.80 Å	2WVI	[15]
C-terminal BUB1	*H. sapiens*	724-1085	2.31 Å	3E7E	[47]

Protein-peptide complexes					
Bub3–GLEBS Bub1	*S. cerevisiae*	315-350 (Bub1); full-length (Bub3)	1.90 Å	2I3S	[31]
Bub3–GLEBS Mad3	*S. cerevisiae*	353-395 (Mad3); full-length (Bub3)	2.80 Å	2I3T	[31]

**Table 2:** Human *BUB1* amino acid substitutions associated with cancer

BUB1 region	Residue	Domain	Clinical condition	Refs
N-terminal	E36→D	TPR domain	Colorectal cancer	[49]
Deletion 76-141, frameshift	Colorectal cancer	[48]
A130→S	Lymph node metastasis	[53]
140, transition of the splicing donor site	Colorectal cancer	[48]
R209→Q	Lung cancer	[57]
G250→N	GLEBS motif region	ATLL	[67]^a^
Y259→C	Pancreatic cancer	[54]
H265→N	Pancreatic cancer	[54]
Middle	S375→F	Low complexity region	Colorectal cancer	[68]
S492→Y		Colorectal cancer	[48]
K566→R		Colorectal cancer	[68]
P648→R		Colorectal cancer	[49]
C-terminal	827 Deletion, frameshift	Kinase domain	Thyroid follicular adenoma	[56]
S950→G	Colorectal cancer	[69]

a These authors incorrectly number these residues; the numbering show here is correct.

**Table 3:** Human *BUBR1* amino acid substitutions associated with cancer

BUBR1 region	Residue	Domain	Clinical condition	Refs
N-terminal	M40→T	KEN box region	Colorectal cancer	[48]
Y155→C	TPR domain	MVA	[40]
E166→D	ATLL	[67]^a^
R224→, nonsense	PCS syndrome	[60]
A302→P	Low complexity region	ATLL	[67]
Q/A349→A/Q	Glioblastomas, breast cancer, colorectal cancer	[49,68]
Q→R	Glioblastomas	[70]
Q363→R	Identified in breast cancer cell lines	[71]
E390→D	Close to the GLEBS motif region	Wilms tumor	[72]
Middle	523-538, deletion	Low complexity region	ATLL	[67]
	R550→Q	Microcephaly, eye abnormality, MVA	[40,73]
	X612 Deletion, frameshift	PCS syndrome	[60]
	V618→A	Colorectal cancer	[74]
	R727→C	MVA	[40]
	738, insertion, frameshift	MVA	[59]
C-terminal	R814→H	Kinase domain	MVA	[40,59]
L844→F	Cryptorchidism	[40,75]
I909→T	Cerebellar hypoplasia, MVA	[40,73]
Q921→H	No observable effects	[40,59]
S928→nonsense	B-cell lymphoma	[67]
L1012→P	Hypothyroidism, anemia	[40,59]
1023, deletion	Colorectal cancer	[48]
L1031→Q	ATLL	[67]

a These authors incorrectly number these residues; the numbering show here is correct.

## Box 1 The mitotic SAC

The function of this self-monitoring mechanism can be summarized as follows: in prometaphase, the nuclear envelope breaks down and microtubules that emanate from opposite poles attach to the kinetochores of individual sister chromatids. Cells with an unsatisfied checkpoint recruit BUB1, BUBR1, BUB3, MAD1, MAD2 and CDC20 to unattached kinetochores (Figure I). MAD1 forms a stable complex with MAD2 *in vitro.* An inactive open-MAD2 conformation is catalytically converted to a closed-MAD2 conformation that is able to bind to CDC20. The MAD2–CDC20 association prevents loss of cohesion on bi-oriented sister chromatids because it triggers the recruitment of BUBR1–BUB3 into an APC/C inhibitory complex. BUBR1 association with BUB3, MAD2, and CDC20 is referred as the MCC, which interacts with the APC/C to render it inactive (Figure I). The function of the SAC is linked to the kinetochore–microtubule network through the physical interaction of BUB1 and BUBR1 with blinkin; this interaction is required for directing BUB1 and BUBR1 to the kinetochores. In metaphase, the checkpoint is satisfied (i.e. all chromosomes undergo bipolar attachment and are aligned at the center of the cell), which releases APC/C–CDC20 inhibition. This is followed by the onset of anaphase, in which sister chromatids separate and are pulled toward opposite poles of the cell. When the checkpoint is satisfied, securin can be ubiquitylated by APC/C and degraded. This leads to the release and activation of separase, a caspase-like protease that cleaves cohesin, the molecule that holds sister chromatids together at the centromere. The cleavage of mitotic cohesin at centromeres and chromosome arms is followed by chromosome separation and mitotic progression from M-phase to interphase. Mitotic exit is achieved by destruction of cyclin B1, which leads to inactivation of CDK1. This is followed by disassembly of the spindle, decondensation of chromosomes and re-assembly of the nuclear envelope.

## Box 2 A model of BUB1 and BUBR1 (Mad3) interactions in the SAC

The 3D structural information of different SAC components provides clues to the function of the SAC. First, when the SAC is unsatisfied, BUB1 and BUBR1 are recruited to unattached or incorrectly attached kinetochores through the formation of a ternary complex with blinkin (Figure I). The BUB1 KEN boxes mediate BUB1 degradation by the APC/C–CDH1 in the G1 phase of the cell cycle and BUB1–CDC20 binding. Phosphorylation of CDC20 S153 by BUB1 is required for efficient SAC activity. The BUBR1 and BUB1 TPR domains each bind blinkin, whereas their C-terminal regions mediate interactions with each other and with BUB3 through the GLEBS motif. Binding to the kinetochore stimulates BUBR1 kinase activity, which results in prolonged mitotic arrest. CENP-E-dependent inhibition of BUBR1 kinase activity probably leads to microtubule attachment that overcomes the mitotic arrest. Second, direct interactions between the BUBR1 KEN boxes and CDC20, when BUBR1 forms part of the MCC, results in the inhibition of APC/C–CDC20 ubiquitin ligase activity. One potential mechanism by which BUBR1 kinetochore recruitment induces prolonged APC/C–CDC20 inhibition posits the existence of a signaling cascade whose downstream targets include enzymes that post-translationally modify BUBR1 (including acetylation of residue K250 by PCAF), thus affecting its interaction with and inhibition of APC/C–CDC20. Alternatively, kinetochore recruitment might induce conformational changes in BUBR1 thereby enabling subsequent APC/C inhibition.

## Glossary

ATLL: a highly aggressive non-Hodgkin's lymphoma of the patient's own T-cells.
Aneuploidy: a condition in which premature separation of sister chromatids results in the loss or gain of chromosomes in daughter cells. Aneuploidy constitutes a prevalent form of genetic instability observed in many types of human cancer.
Blinkin: a human protein that acts as a central component of the Knl1–Mis12–Ndc80 (KMN) network. In humans it is also referred to as KNL1, CASC5 and AF15Q14; known as KNL-1 in worms, Spc105 in yeast and Spc105R in flies.
CENP-E: a member of the kinesin motor protein family that accumulates in the G2 phase of the cell cycle and first appears at the centromere region of chromosomes during prometaphase.
CENP-F: a protein that associates with the centromere–kinetochore complex during mitosis. This protein maintains its association with the kinetochore throughout early anaphase and binds CENP-E and BUB1, thus suggesting a role in chromosome segregation and/or the mitotic checkpoint.
Knl1–Mis12–Ndc80 (KMN) network: A conserved assembly consisting of Knl1, the Mis12 complex, and the Ndc80 complex that constitutes the core attachment site for microtubules at the kinetochore and recruits components that generate the mitotic checkpoint signal. The Mis12 complex is formed by the proteins Mis12, Dsn1, Nnf1 and Nsl1 whereas the Ndc80 complex is formed by Ndc80, Nuf2, Spc24 and Spc25.
Paralog: homologous sequences that are the result of a gene duplication event and have descended side by side during the history of an organism.
P+1 loop: a small motif, residing immediately downstream of the activation loop of a kinase domain. The P+1 loop received its named for its role in contacting the residue immediately C-terminal to the phosphorylated tyrosine (the P+1 position of the residue) in the substrate. The P+1 loop is implicated in recognizing the residues next to tyrosine to be phosphorylated in the substrate.
TPR motif: a protein motif defined by a helix–loop–helix, in which the consensus 34 amino acids sequence contains small hydrophobic residues at positions 8, 20 and 27; large hydrophobic residues at positions 4, 7, 11 and 24; and one α-helix-breaking residue, typically a proline, at position 32.
